# Differential activity of methylene blue against erythrocytic and hepatic stages of *Plasmodium*

**DOI:** 10.1186/s12936-018-2300-y

**Published:** 2018-04-03

**Authors:** Henriette Bosson-Vanga, Jean-François Franetich, Valérie Soulard, Daniel Sossau, Maurel Tefit, Bocar Kane, Jean-Christophe Vaillant, Steffen Borrmann, Olaf Müller, Nathalie Dereuddre-Bosquet, Roger Le Grand, Olivier Silvie, Dominique Mazier

**Affiliations:** 1 Sorbonne Université, Inserm, CNRS, Centre d’Immunologie et des Maladies Infectieuses, U1135, ERL8255, CIMI-Paris, F-75013 PARIS, France; 2 Département de Parasitologie-Mycologie, UFR des Sciences Pharmaceutiques et Biologiques, Université Félix Houphouët Boigny, Abidjan, Côte d’Ivoire; 3 Department of Dermatology, Eberhard Karls University, Tübingen, Germany; 4 UPMC, UMS28, 105 Bd de l’hôpital, 75013 Paris, France; 5 Service de Chirurgie Digestive, Hépato-Bilio-Pancréatique et Transplantation Hépatique, AP-HP, Groupe Hospitalier Pitié-Salpêtrière, 83 Bd de l’hôpital, 75013 Paris, France; 6 German Center for Infection Research (DZIF), Tübingen, Germany; 7 Institute for Tropical Medicine, University of Tübingen, Tübingen, Germany; 8 Institute of Public Health, Medical School, Ruprecht-Karls-University, Heidelberg, Germany; 9 CEA, INSERM U1184, Immunology of Viral Infections and Autoimmune Diseases, Université Paris Sud 11, Fontenay-aux-Roses, France; 10 Service de Parasitologie-Mycologie, Centre National de Référence du Paludisme, AP-HP, Groupe Hospitalier Pitié Salpêtrière, 83 Bd de l’hôpital, 75013 PARIS, France

**Keywords:** Malaria, Methylene blue (MB), Exoerythrocytic stages, *Plasmodium*, Blocking malaria transmission

## Abstract

**Background:**

In the context of malaria elimination/eradication, drugs that are effective against the different developmental stages of the parasite are highly desirable. The oldest synthetic anti-malarial drug, the thiazine dye methylene blue (MB), is known for its activity against *Plasmodium* blood stages, including gametocytes. The aim of the present study was to investigate a possible effect of MB against malaria parasite liver stages.

**Methods:**

MB activity was investigated using both in vitro and in vivo models. In vitro assays consisted of testing MB activity on *Plasmodium falciparum*, *Plasmodium cynomolgi* and *Plasmodium yoelii* parasites in human, simian or murine primary hepatocytes, respectively. MB in vivo activity was evaluated using intravital imaging in BALB/c mice infected with a transgenic bioluminescent *P. yoelii* parasite line. The transmission-blocking activity of MB was also addressed using mosquitoes fed on MB-treated mice.

**Results:**

MB shows no activity on *Plasmodium* liver stages, including hypnozoites, in vitro in primary hepatocytes. In BALB/c mice, MB has moderate effect on *P. yoelii* hepatic development but is highly effective against blood stage growth. MB is active against gametocytes and abrogates parasite transmission from mice to mosquitoes.

**Conclusion:**

While confirming activity of MB against both sexual and asexual blood stages, the results indicate that MB has only little activity on the development of the hepatic stages of malaria parasites.

## Background

Malaria remains a major cause of morbidity and mortality in many regions of the world [1]. In 2016 the World Health Organization estimated a total of 212 million cases of malaria, which resulted in 429,000 deaths. African populations, particularly children younger than 5 years old, are the most affected by this disease [2]. Human malaria is caused by five different species of *Plasmodium* parasites. *Plasmodium falciparum* and *Plasmodium vivax* are the most common forms with *P*. *falciparum* being the deadliest. Due to the ability to rapidly develop drug resistance, *Plasmodium* parasites continue to be a major challenge for effective case management [3, 4]. Malaria parasites consist of several life cycle stages that have to be individually targeted to reach malaria elimination [5].

Most of the drugs used for the treatment of malaria act as erythrocytic stage inhibitors. The ambitious goal of malaria elimination, however, requires strategies to prevent parasite transmission between the human host and the mosquito vector, thus targeting the hepatic or the gametocyte developmental stages [6]. To date, primaquine (PQ) is the only approved compound active against all stages of *Plasmodium* development: liver stages, including hypnozoites, and blood stages, including gametocytes, thereby also blocking parasite transmission to mosquitoes. However, PQ causes haemolytic anaemia in moderate to severely glucose-6-phosphate dehydrogenase (G6PD)-deficient individuals [7]. Moreover, recent studies show that PQ is ineffective in people with low metabolizing cytochrome P450 2D6 genotypes [8]. Attempts to develop a replacement for PQ led to tafenoquine, a drug of the same class with partly more favourable pharmacokinetics, but which is still deleterious in patients with G6PD deficiency [9, 10]. PQ was initially derived from methylene blue (MB) [11, 12], the first synthetic drug ever used against malaria [13]. MB is a subversive substrate and specific inhibitor of the *P. falciparum* disulfide reductase. It played a major role in malaria treatment before and during World War II [14]. In recent years, there was a surge of interest in MB as an anti-malarial agent when *P. falciparum* glutathione reductase was identified as a new drug target [15]. In vitro MB is active against parasites resistant to standard drugs [16], and oral MB was used to treat uncomplicated malaria in Burkina Faso [17]. A triple combination of MB with artesunate and amodiaquine was shown to be effective against the gametocytes of *P. falciparum* [18]. Furthermore, these clinical trials in Africa showed that effective antiplasmodial doses of MB are safe in both adults and children including G6PD-deficient individuals [19]. MB appears to be a potentially useful partner drug for existing artemisinin-based combination therapy (ACT), which is first-line anti-malarial treatment. On the contrary to its well-described inhibitory activity on blood stages, whether MB is able to interfere with *Plasmodium* liver stages is still an open question. Here, MB’s activity was assessed against *Plasmodium spp* liver stages, including hypnozoites, in vitro and in vivo, and its transmission blocking activity was analysed in vivo.

## Methods

### Drugs and chemicals

**Fig. 1:**
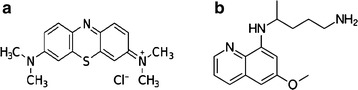
Chemical structures of **a** methylene blue (MB) and **b** primaquine (PQ)

The chlorure of methylthioninium Proveblue was used in this study. It is a MB preparation provided by Provepharm; a company specialized in the development of pharmaceutical products. Today, Proveblue^®^ methylene blue active pharmaceutical ingredient (API) is fully compliant with most recent pharmacopoeial standards on MB. Proveblue was obtained from Olaf Müller. Figure 1 shows the chemical structure of the compound. For in vitro and in vivo assays MB was dissolved in distilled water. Primaquine used in these studies was obtained from Sigma-Aldrich, USA. Stock solutions were prepared at 10 mg/ml in distilled water. d-Luciferin, provided by OZBioscences, a substrate for luciferase was dissolved in 1× phosphate-buffered saline (PBS) (Gibco) and injected intraperitoneally at 100 mg/kg.

### Parasites and sporozoites isolation

Three species of *Plasmodium* were used in this study:

1. *Plasmodium falciparum* (NF54 strain) sporozoites were obtained from infected salivary glands of *Anopheles stephensi* 14–21 days after an infective blood meal (Department of Medical Microbiology, Radboud University Medical Center, Nijmegen, The Netherlands).
2. *Plasmodium cynomolgi* (M strain) sporozoites were obtained from infected salivary glands of *An. stephensi* 14–16 days after an infective blood meal (blood infected with *P. cynomolgi* was obtained from *Macaca fascicularis*, the natural host of *P. cynomolgi*, infected with cryopreserved blood stages) (CEA, Fontenay aux Roses, France).
3. *Plasmodium yoelii* (17XNL strain), line expressing GFP-Luciferase (GFP-luc) was generated using a ‘Gene Out Marker Out’ strategy [20].

Infected salivary glands were aseptically removed by hand dissection, crushed in a potter and filtered through a 40-µm filter for sporozoite isolation (Cell Strainer, BD BioSciences, USA). Sporozoites were counted using a disposable Glasstic microscope slide (KOVA, USA).

### Mice and mosquitoes

Six to 8 weeks old female BALB/c mice (18–22 g), obtained from Janvier (Le Genest-Saint-Isle, France) were used. For in vivo transmission blocking studies with *P. yoelii*, 4–5 days old *An. stephensi* female mosquitoes were used as experimental vectors.

### Hepatocytes

Primary human hepatocytes were isolated from liver segments obtained from adult patients undergoing partial hepatectomy (*Service de Chirurgie Digestive, hépato*-*bilio pancréatique*, *Hôpital Pitié Salpêtrière*, Paris France). Primary simian hepatocytes were isolated from liver segments collected from healthy *Macaca fascicularis* from CEA, Fontenay aux Roses, France or ICM, *Hôpital Pitié Salpêtrière*, Paris France. Primary murine hepatocytes were isolated from liver segments collected from BALB/c mouse.

All hepatocytes were isolated using collagenase perfusion as previously described [21, 22]. Simian hepatocytes were immediately cryopreserved with a Nicool-Freezal (Air liquide Santé, Marne la vallée) and then used when needed after fast thawing at 37 °C. Human and murine hepatocytes were used in fresh cultures. Cells were seeded in 96-well plates (Falcon by Becton–Dickinson Labware Europe, France) coated with collagen I (BD Biosience, USA), at a density of 80,000 cells per well. Human, simian and murine hepatocytes were maintained at 37 °C in 5% CO_2_ in William medium (Gibco) supplemented with 10% of fetal bovine serum (FBS, FetalClone, Hyclone), 1% penicillin–streptomycin (Gibco), 5 × 10^−3^ g/l insulin (Sigma Aldrich, USA) and 5 × 10^−5^ hydrocortisone (Upjohn Laboratories SERB, France) until infection with sporozoites. For human hepatocytes, the complete medium was supplemented with 2% dimethyl sulfoxide (DMSO, Sigma- Aldrich, USA) until infection.

### Assessment of liver stage development in vitro

Hepatocytes were seeded into collagen-coated black 96-wells plates and maintained at 37 °C in 5% CO_2_ in a complete medium. On the day of infection, 3 × 10^4^ sporozoites of *P. falciparum*, *P. cynomolgi* or *P. yoelii* resuspended in complete medium were added to the respective host cell cultures (human, simian or murine). The infected culture plates were centrifuged for 10 min at 900*g* allowing fast parasite sedimentation and further incubated for 3 h at 37 °C/5% CO_2_ allowing parasites invasion into the hepatocytes. Serial dilution of the tested drugs, MB and PQ were added to the cultures. Culture medium containing the appropriate drug concentration was changed daily and cells were fixed 48 h post infection (pi) for *P. yoelii*, 6 days pi for *P. falciparum* or 8 days pi for *P. cynomolgi*.

### Parasite counting and schizont size determination using high-content imaging

The number, size and shape of the parasites were determined using a CellInsight High Content Screening platform equipped with the Studio HCS software (ThermoFisher Scientific). Parasite immunostaining was done using a α-*Plasmodium* HSP70 antibody raised in mice and a secondary anti-mouse antibody coupled to AlexaFluor 488 [23]. Host cell and parasite nuclei were labelled with 4′,6-diamidino-2-phenylindole (DAPI). Thirty-seven images, representing more than 95% of the total bottom surface area of a culture well in 96-well plates, were captured for analysis. The custom script used for counting and measuring parasites first required identification of the objects on the basis of a fluorescence intensity threshold. The object identity was then further validated based on size and morphological (shape) criteria. The presence of DAPI-associated fluorescence in the selected objects allowed for their final selection and rejection of false positives. Inhibitory concentration 50 (IC_50_) value is the drug concentration at which a 50% reduction of the exo-erythrocytic forms (EEFs) number was observed, as compared to the control cultures. IC_50_ were calculated using ICEstimator software version 1.2 [24].

The parasite size reduction is calculated on the average object area using the total surface area of each selected object (µm^2^). Cell confluence for primary hepatocytes was measured by counting the absolute number of host cell nuclei using a script that first requires identification of the objects on the basis of the DAPI fluorescence intensity threshold and then involved a validation step based on size and morphological (shape) criteria. The objects on the edge of the pictures were excluded. The toxic concentration 50 (TC_50_) value was determined as the drug concentration at which a 50% reduction of the cell confluence was estimated. With IC_50_ and TC_50_, therapeutic index (TI) was calculated (TC_50_/IC_50_).

### Assessment of liver stage in vivo development

Five mice per treatment group were used. Experimental groups were treated with MB at 50 and 100 mg/kg by intra-peritoneal (ip) injection. Untreated infected mice, non-infected mice (infection controls) as well as PQ-treated (50 mg/kg) mice (control drug) were also included. The drugs were administrated on days − 1, 0, +1, and mice were challenged on day 0 by retro-orbital injection of *P. yoelii* (GFP-luc strain) sporozoites (10,000 per mouse). In vivo imaging was performed 44 h post-infection to assess liver stage development. When parasites were detected in the liver, treatment was continued until day 5 to assess blood stage activity. On day 4 and 6 pi, in vivo imaging and GIEMSA-stained blood smears were performed to monitor blood stage development using methods described previously [25]. IVIS Spectrum (Caliper Life Science, Hanover, MD, USA) was used to measure luciferase activity. Prior to analysis the mice were injected ip with D-luciferin (100 mg/kg), anesthetized with isoflurane and imaged 10 min post-injection. Images were analysed using the living Image 3.0 software (Capiler Life Science, Hanover, MD, USA). For blood stage assessment, luminescence (total flux photons/seconde) and Giemsa-stained blood smears were done on days 3, 4 and 6 pi to evaluate the blood stage prepatent period in mice after sporozoite inoculation.

### In vivo blood stage and transmission blocking activity assays

Mice were infected by ip inoculation of 10^7^ erythrocytes parasitized with GFP-luc *P*. *yoelii* and separated in two groups. The first group was treated from day 0 to day 3. For the second group, treatment was initiated 4 days after the infection, 2 h before mosquito feeding [26]. For each compound (Control: PBS, MB 50 mg/kg) and for each treatment protocol, mice were randomly separated into groups of five animals. At day 3, the presence of gametocytes was checked by microscopic analysis of Giemsa-stained blood films and their functionality was assessed using microgamete exflagellation assay. Mice were anesthetized and placed according to the treatment on the top of individual cages containing 100 glucose-starved *An. stephensi* female mosquitoes, which were allowed to feed for 1 h. Unfed mosquitoes were removed from the cage. Seven days after the blood meal, 30–40 mosquitoes were dissected and their midguts examined under light microscope (400×) to assess oocyst presence. Two weeks after the blood meal, salivary glands of 30–40 mosquitoes per group were dissected to determine the average number of sporozoites per mosquito in each group. Inhibition activity was determined by determining the percentage of oocyste-positive mosquitoes (infection rate), the mean number of oocysts per mosquito (oocyst burden) and the mean number of sporozoites per mosquito.

### Statistical analysis

Excel 2007 spreadsheet (Microsoft office) and GraphPad Prism 6 statistical Software (GraphPad. Software, San Diego, CA, USA) were used for data analysis. All values were expressed as means and standard deviations (SD). A *p* value of 0.05 or less was considered as statistically significant.

## Results

### In vitro activity against *Plasmodium* liver stages

**Fig. 2:**
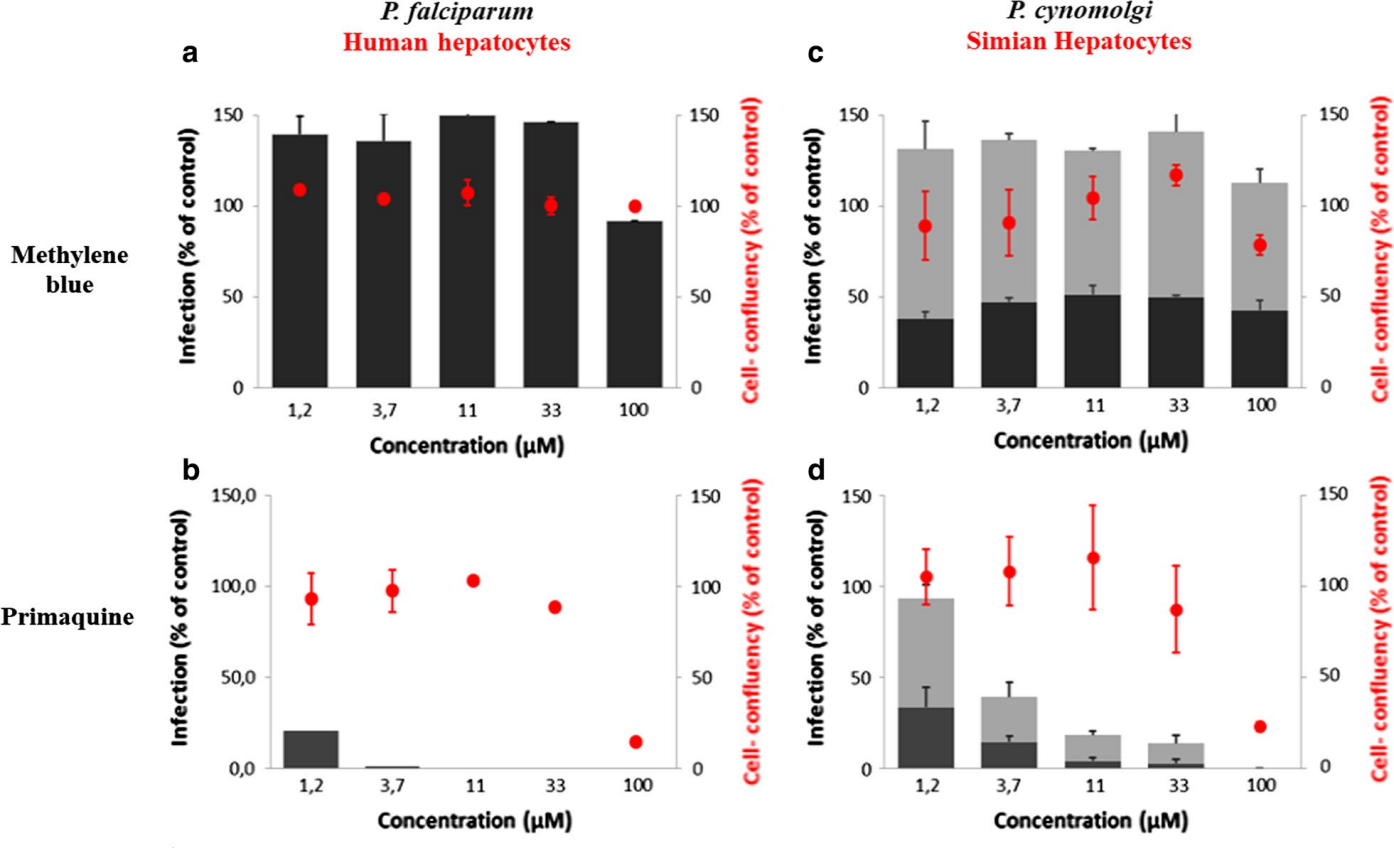
Activity of methylene blue (MB) and primaquine (PQ) in primary hepatocytes. **a** Activity of MB against liver stage of *P. falciparum* in human hepatocytes, (N 221 ± 50). **b** Activity of PQ against liver stage of *P. falciparum* in human hepatocytes, (N 301 ± 55). **c** Activity of MB against liver stage of *P. cynomolgi* in simian hepatocytes, (N 176 ± 22). **d** Activity of PQ against liver stage of *P. cynomolgi* in simian hepatocytes, (N 237 ± 4). Activity (infection scale, black bars = EEFs, grey bars = hypnozoites) and toxicity to host cells (cell confluency scale and red circles). Data are mean ± SD of triplicate measurements from a single representative experiment. *N* number of parasites in control

**Fig. 3:**
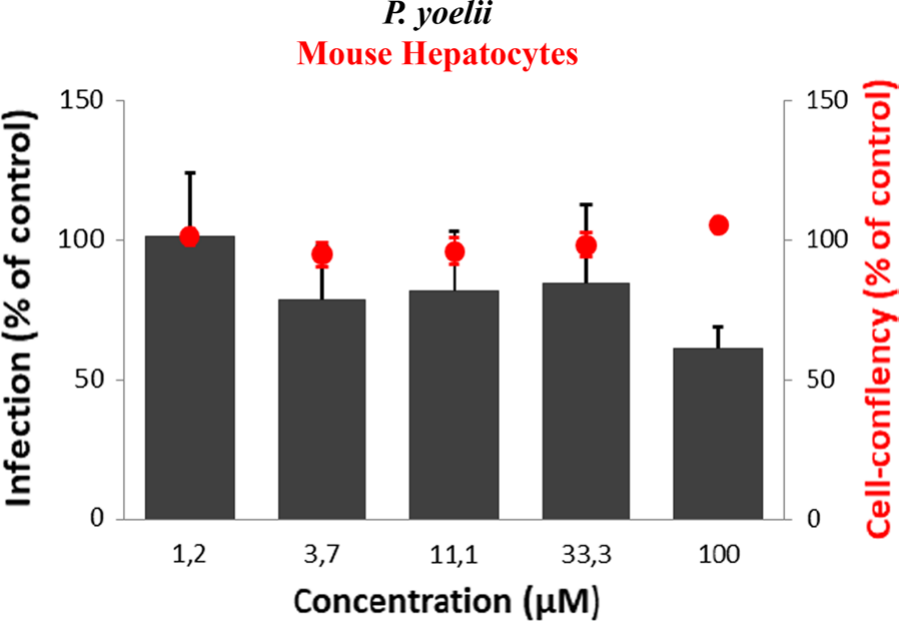
Activity of methylene blue against *Plasmodium yoelii* in murine primary hepatocytes. (N 34 ± 6). Activity (infection scale, black bars = EEFs, grey bars = hypnozoites) and toxicity to host cells (cell confluency scale and red circles). Data are mean ± SD of triplicate measurements from a single representative experiment

MB was tested at concentrations ranging from 0.02 to 100 µM on primary murine, human and simian hepatocytes infected, respectively, with *P. yoelii, P. falciparum* and *P. cynomolgi*. MB and PQ (Fig. 1) cytotoxicity was evaluated by assessing cell confluence and number of DAPI-positive hepatocytes after drug treatment and anti-malarial activity was evaluated by counting EEFs (Figs. 2 and 3).

As shown in Table 1, MB had no effect (IC_50_ > 100 µM) on *P. falciparum*, *P. cynomolgi* and *P. yoelii* liver stages including *P. cynomolgi* hypnozoites (Fig. 2c). The corresponding TI (TI = TC_50_/IC_50_) in these primary hepatocytes (TC_50_ > 100 µM) was very low (TI = 1). As expected, PQ was highly active on *P.* *falciparum* and *P. cynomolgi* liver stages with IC_50_ of 0.6 µM in human cells and 2.72 µM in simian cells. The TI of PQ was, respectively, 66.5 in human hepatocytes (TC_50_ 39.93 ± 2.29 µM) and 14.22 in simian hepatocytes (TC_50_ 38.7 ± 2.98 µM). In addition, MB had limited effect on parasite development, as evaluated by measuring parasite size (Table 1). At 11 µM, MB induced a 10% reduction of *P. falciparum* and *P. cynomolgi* parasite area.

**Table 1:** Comparative inhibitory activities of Proveblue and primaquine on *Plasmodium yoelii*, *Plasmodium falciparum* and *Plasmodium cynomolgi*

Drugs	Methylene blue			Primaquine		
Parasites	IC_50_ (µM)	TI	Size reduction at 11 µM (%)	IC_50_ (µM)	TI	Size reduction at 11 µM (%)
*P. yoelii* (MH)	> 100	1	10	0.64 ± 0.1^a^	110^a^	ND
*P. falciparum* (HH)	> 100	1	10	0.6 ± 0.06	66.5	100
*P. cynomolgi* (SH)	> 100	1	10	2.72 ± 0.34	14.22	70

*MH* mouse hepatocytes; *HH* human hepatocytes; *SH* simian hepatocytes; *TI* therapeutic index: TC_50_/IC_50_, *ND* not determined

^a^[51]

### In vivo activity against *Plasmodium yoelii* liver stage

**Fig. 4:**
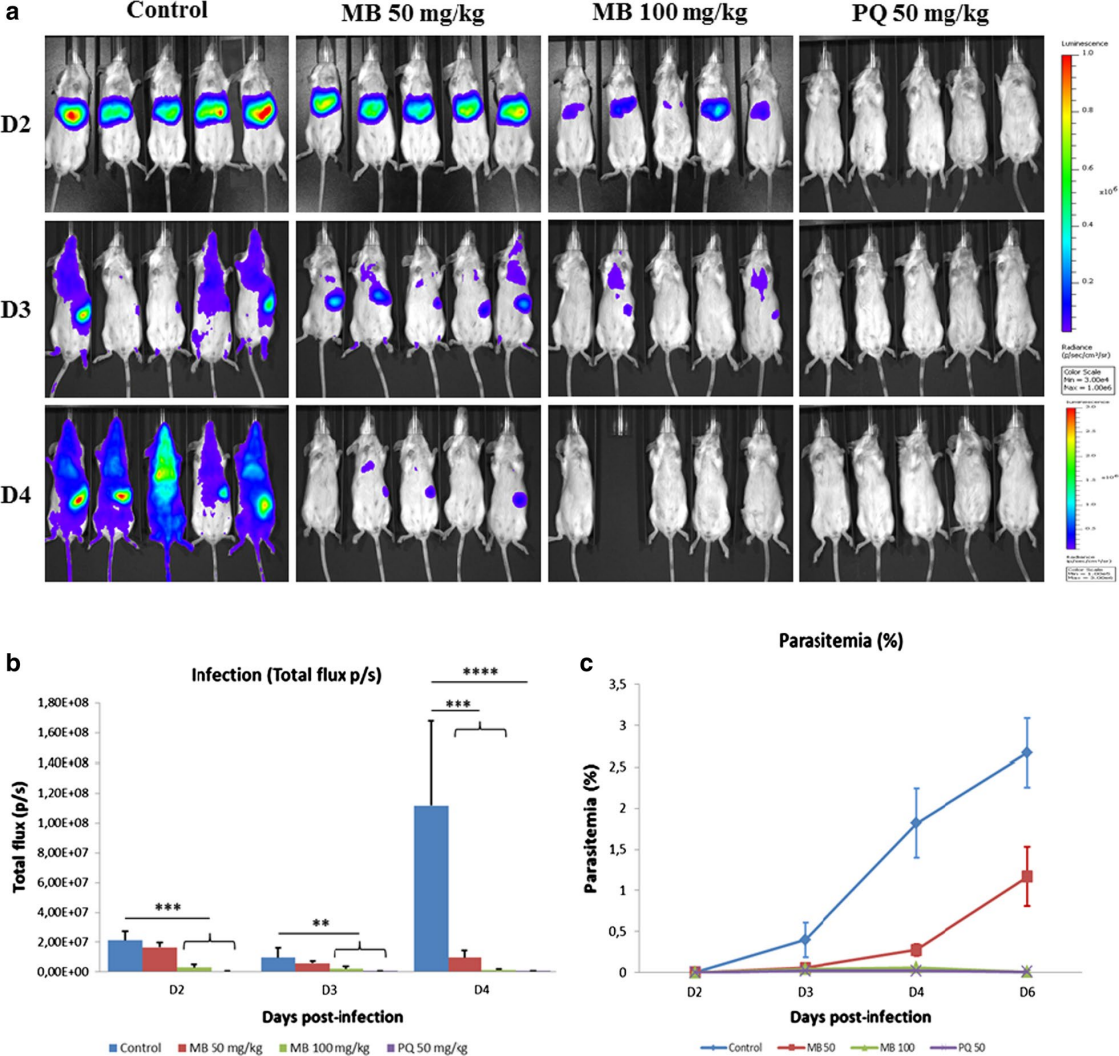
*In vivo* anti-malarial activity of methylene blue against liver and blood stage. Mice were infected with 10,000 sporozoites. MB (50 and 100 mg/kg) and PQ (50 mg/kg) were administreted on day − 1, 0 and 1 to monitor activity against liver stage on day 2 and on day 3 and 4 to monitor blood stage activity. **a** Representative in vivo images (IVIS) of luminescence show in the liver of live Balb/C mice at different time points. Rainbow images show the relative levels of luminescence ranging from low (blue), to medium (green), to high (yellow/red). **b** Mean luminescence levels (photons/sec) of each group at day 2, 3 and 4; (**p = 0.0031, ***p = 0.0001, ****p < 0.0001). **c** Blood stage patency was monitored in mice by Giemsa-stained blood smears on day 3, 4 and 6. The results are expressed as the mean ± standard errors of the mean of 4 or 5 measurements

Two groups of BALB/c mice were treated with MB at 50 and 100 mg/kg or PQ at a concentration of 50 mg/kg one day prior the infection, the day of infection and 1 day post-infection. PQ (50 mg/kg) served as a positive control. The results illustrate that MB was unable to prevent parasite blood stage development (Fig. 4a). Mice treated with PQ were free of parasites throughout the infection experiment (Fig. 4a). Mean luminescence (photons/sec) values collected from liver location of mice were 2.11 × 10^7^ (± 6.39 × 10^6^) for untreated control group, 1.66 × 10^7^ (± 3.07 × 10^6^) for MB 50 mg/kg and 2.97x10^6^ (± 2.00 × 10^6^) for MB 100 mg/kg at 44 h pi (Fig. 4b). *Plasmodium yoelii* liver stage inhibition was, respectively, 21.43 and 85.9% for MB at 50 and 100 mg/kg. At days 3 and 4 post-infection, the luminescence signal spread in the entire body due to parasite dissemination in the blood circulation. When the growth of parasites is compared between day 2 and day 4, the signal increased five times in the control group whereas in the groups treated with MB there was a reduction of the luminescence signal (Fig. 4b). In order to validate these results, mean luminescence was correlated with blood parasitaemia based on blood smears microscopic examination on days 3, 4 and 6 pi. Compared to untreated controls, blood parasitaemia was strongly diminished after treatment of mice on days 4 and 6 with 50 and 100 mg/kg MB. The protocol using 100 mg/kg showed a slightly increased effectiveness (Fig. 4c). These results show that MB has a moderate effect on liver stages, albeit at a level unable to prevent blood stage development. Conversely, MB strongly inhibits blood stage growth in vivo.

### In vivo transmission blocking assays

**Fig. 5:**
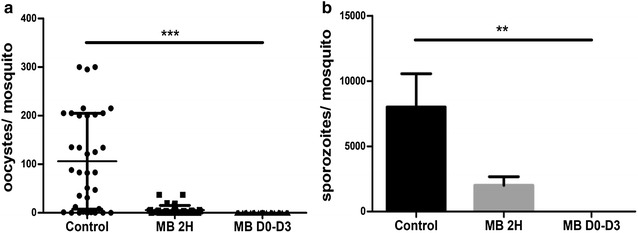
Impact of methylene blue 50 mg/kg on sporogonic development. **a** Number of oocysts per midgut. Each point represents the oocyst number from an individual mosquito. Oocysts density in MB groups significantly different with the untreated group (***p < 0.0001). **b** Average of sporozoites extracted from salivary glands of mosquitoes (Total of sporozoites/number of mosquitoes). (**p < 0.0016)

In order to analyse the ability of MB to inhibit parasite transmission to mosquitoes, *An. stephensi* mosquitoes were fed on *P. yoelii*-infected BALB/c mice with or without MB treatment. The mean number of oocysts per mosquito (oocyst burden) and the mean number of sporozoites per mosquito were dramatically decreased showing that MB efficiently reduces transmission (Fig. 5). Table 2 shows the mean parasitaemia and gametocytaemia before a mosquito’s blood meal. Mice treated from day 0 to day 3 showed a remarkable reduction of parasitaemia of nearly 79% as well as a reduction of gametocytaemia of 100% compared to the control group. These results clearly show that MB can inhibit parasite transmission by eliminating gametocytes. Treatment applied 2 h before mosquito feeding had no effect on the gametocyte rate in the mice, as observed on blood smears, but induced a drastic reduction of both the mosquito infection rate and the number of oocysts in infected mosquito midguts dissected 1 week post infection (mean number of 128 oocysts per infected mosquito in the control group compared to 8 oocysts in the treated group). As expected, this decrease in the oocyst number led to a strong reduction in sporozoite numbers in salivary glands 2 weeks post mosquito blood meal (Fig. 5b). From this last experiment, the conclusion was that a short-term exposure to MB is sufficient to prevent *Plasmodium* transmission to *An. stephensi* mosquitoes.

**Table 2:** Parasitaemia and gametocytaemia before feeding and prevalence of infected mosquitoes in each group

	Untreated	MB 2H	MB D0–D3
Parasitaemia (%)	0.95 ± 0.39	1.36 ± 0.18	0.2 ± 0.02
Gametocytaemia (%)	0.08 ± 0.02	0.097 ± 0.06	0
Prevalence infected mosquitoes (%)	82.28 (29/35)	65.71 (23/35)	0 (0/35)
Oocytes mean per infected mosquitoes	128	8	0

The results are expressed as mean ± standard errors. The result shown is representative of 1 assay of the 2 independent assays performed

## Discussion

Most currently available anti-malarial drugs primarily target the disease-causing parasite stages in the human blood system. However, to eradicate malaria, new drugs that block transmission of the parasite between the human host and the mosquito vector, and eliminate the various stages of the parasite during its cycle in the human body, are needed. It is surprising that few studies [27], to date, have examined drug activity against liver, sexual blood and mosquito stages of the malaria parasite at the same time. In this study, PVB activity was assessed on these different parasite stages. PVB is a MB preparation that complies with the European Pharmacopoeia, contains limited organic impurities and heavy metals of recognized toxicity, and has previously been demonstrated to possess anti-malarial activity on asexual and sexual blood stages. In vitro experiments confirmed PVB anti-malarial potency against the blood stage of 23 *P. falciparum* strains that were resistant to various anti-malarial drugs [16]. In combination with mefloquine, quinine and dihydroartemisinin, PVB showed synergistic effects in vitro against 9 well-established *P. falciparum* strains [28, 29]. Ex vivo experiments on 19 samples isolated from patients showed IC_50_ for PVB ranging from 0.88 to 40.2 nM with a mean of 5.3 nM [30]. PVB treatment at 1–10 mg/kg for 5 days significantly reduced or prevented cerebral malaria in mice [31–33]. In humans, several clinical trials conducted in Burkina Faso showed that oral MB was safe and effective in the treatment of uncomplicated falciparum malaria when combined with other anti-malarials [17, 18, 34–36]. Another study reported moderate curative activity with MB monotherapy, illustrating the need for this slow-acting drug to be combined with fast-acting anti-malarials [37]. Another advantage of MB is that it has gametocytocidal properties and can reduce the transmission of *P. falciparum*. In fact, MB interferes in vitro on gametocyte development at all stages and can block transmission through clearance of stage V gametocytes [38]. Previous clinical observations showed that MB reduced gametocyte carriage rate in treated patients [18, 39]. So far no published study has analysed the activity of MB on the hepatic stage of *Plasmodium* parasites. Here, several established models of malaria liver stages were used to evaluate the effectiveness of PVB. The *P. yoelii* murine model has two main interests, it allows: (1) a quick and ‘easy’ in vitro screening of the molecules; and, (2) to draw a parallel between in vitro and in vivo experiments. Murine *Plasmodium* species are commonly used to study the liver stage of infection in mice and in vitro, yet it is not clear how well these models reflect *P. falciparum* infections in humans. Then, two in vitro models: *P. falciparum* and *P. cynomolgi* were used to validate the results with *P. yoelii* model. The *P. falciparum* experiments provide direct data on pharmacodynamic effects against the liver and blood stages of this most important human malaria parasite. The *P. cynomolgi* system is being used as a model for *P. vivax* and replicates its capability to produce hypnozoites. This *P. cynomolgi* model has been used for decades to identify radical curative compounds against *P. vivax* [40, 41]. In all these models, PVB showed no significant activity on *Plasmodium* liver stage infection in vitro. Interestingly, MB could partially reduce the parasite burden in the liver of *P. yoelii*-infected mice; however this effect was observed only at the highest doses and was not sufficient to prevent transition to a blood stage infection. To date, there is only a limited number of therapeutics capable of eliminating *Plasmodium* liver stages [42, 43]. Whereas PQ, as well as tafenoquine, and atovaquone actively eliminate dividing *Plasmodium* liver stages, only PQ has a pronounced effect on the hypnozoites [22]. However, because of its potential to cause life-threatening acute intravascular haemolysis in individuals with severe G6PD deficiency, PQ cannot be widely used [44]. Thus, the development of new compounds with hypnozoitocidal activity suitable for mass administration has become a priority in the current drive to eliminate malaria.

In the present study, MB shows low or no activity in vitro in primary hepatocytes while PQ is very effective. Next, in vivo experiments were performed to evaluate the effect of MB on hepatic and blood stages development. In vivo imaging of *Plasmodium* growth was used to measure the specific inhibition of parasite hepatic stage development and its consequences on blood stage spreading [45, 46]. All control mice used showed a strong luminescence signal at 44 h, as well as on day 3 and 4 post-infection while luminescence signal was abrogated in animals treated with PQ (50 mg/kg). In these experiments, while MB was not able to prevent spreading of the parasites into the central circulation (Fig. 4), an inhibitory effect was observed using the high dose of 100 mg/kg, demonstrating once again that to prevent erythrocytic infection, hepatic inhibition has to be close to 100%. These results have to be considered with those obtained by Garavito in his thesis [47]. Studying the pharmacological effects of MB, he observed a slight delay in the appearance of the erythrocytic infection.

Finally, the activity of MB against *P. yoelii* blood stages was confirmed and its transmission blocking activity too. Indeed, parasitaemia was reduced by 80% in mice infected with 10^7^ *P. yoelii* infected red blood cells when MB (50 mg/kg) was applied from day 0 to day 3 (Table 2), leading to the disappearance of gametocytes from the circulation. These results are in agreement with preliminary clinical data showing a complete elimination of asexual stages and gametocytes in the blood stream of MB-treated malaria patients [18, 39]. Even more interesting, MB considerably decreased the prevalence of infected mosquitoes when the treatment was administrated 2 h before feeding. These observations are in line with results on gametocyte development in the *P. falciparum* model [38].

Why is MB little effective at the hepatic level while it is very active on blood stage and gametocytes? A failure of its uptake into the infected host cell? The typical example is fosmidomycin, an antibiotic with relatively rapid action against asexual blood stages. Previous work established that fosmidomycin is efficiently transported into *P. falciparum*-infected erythrocytes through parasite-induced new permeability pathways. In contrast, *Plasmodium berghei* liver-stage parasites were shown to be resistant to fosmidomycin, presumably due to the inability of this compound to enter hepatocytes [48–50].

Another explanation could be that, similarly to PQ [8], MB would require metabolization by hepatic cytochromes to become active. This would imply that the effect of MB on *Plasmodium* blood stages is due to one or more active metabolites. This is the case of several drugs that are considered prodrugs. For instance, proguanil is a prodrug and its cyclisation by cytochrome P450 into pharmacologically active triazine metabolite (cycloguanil) inhibits plasmodial dihydrofolate reductase. This biotransformation is catalyzed by CYP2C19 and many anti-malarial lead compounds are being designed and synthesized to exploit this pathway [51].

## Conclusion

This study confirms that MB, a precursor of PQ, belongs to the large group of anti-malarial compounds active against malaria blood-stage parasites. Moreover it has an effect on gametocytes, thus preventing transmission to mosquitoes. Unfortunately, MB, in the formulation used in endemic areas, is likely inactive against *Plasmodium* hepatic stage.

## Abbreviations

DAPI: 4′,6-Diamidino-2-phénylindole
MB: methylene blue
PQ: primaquine
PVB: Proveblue
G6PD: glucose-6 phosphate dehydrogenase
TI: therapeutic index
IC_50_: drug concentration that kill 50% of parasites
TC_50_: drug concentration that kill 50% of the hepatocytes
EEFs: exo-erythrocytic forms
